# Maternal and Antenatal Risk Factors for Stillbirths and Neonatal Mortality in Rural Bangladesh: A Case-Control Study

**DOI:** 10.1371/journal.pone.0080164

**Published:** 2013-11-07

**Authors:** Aatekah Owais, Abu Syed Golam Faruque, Sumon K. Das, Shahnawaz Ahmed, Shahed Rahman, Aryeh D. Stein

**Affiliations:** 1 Department of Epidemiology, Rollins School of Public Health and Laney Graduate School, Emory University, Atlanta, Georgia, United States of America; 2 International Center for Diarrheal Disease Research, Bangladesh, Dhaka, Bangladesh; 3 CARE Bangladesh, Dhaka, Bangladesh; 4 Hubert Department of Global Health, Emory University, Atlanta, Georgia, United States of America; University of Massachusetts Medical School, United States of America

## Abstract

**Objective:**

To identify maternal and antenatal factors associated with stillbirths and neonatal deaths in rural Bangladesh.

**Study Design:**

A prospective cohort study is being conducted to evaluate a maternal and child nutrition program in rural Bangladesh. Cases were all stillbirths and neonatal deaths that occurred in the cohort between March 7, 2011 and December 30, 2011. Verbal autopsies were used to determine cause of death. For each case, four controls were randomly selected from cohort members alive at age 3-months. Multivariable logistic regression was used to identify factors associated with these deaths.

**Results:**

Overall, 112 adverse pregnancy outcomes (44 stillbirths, 19/1,000 births; 68 neonatal deaths, 29/1,000 live births) were reported. Of the stillbirths 25 (56.8%) were fresh. The main causes of neonatal death were birth asphyxia (35%), sepsis (28%) and preterm birth (19%). History of bleeding during pregnancy was the strongest risk factor for stillbirths (adjusted odds ratio 22.4 [95% confidence interval 2.5, 197.5]) and neonatal deaths (adjusted odds ratio 19.6 [95% confidence interval 2.1, 178.8]). Adequate maternal nutrition was associated with decreased risk of neonatal death (adjusted odds ratio 0.4 [95% confidence interval 0.2, 0.8]).

**Conclusions:**

Identifying high-risk pregnancies during gestation and ensuring adequate antenatal and obstetric care needs to be a priority for any community-based maternal and child health program in similar settings.

## Introduction

A comprehensive analysis of the causes of stillbirth in low- and middle-income countries concluded that lack of maternal education, high or low parity, older maternal age, and lack of access to antenatal care were significantly associated with increased risk of stillbirth [1]. Other risk factors for stillbirth include maternal nutritional status, congenital anomalies and infections during pregnancy [2,3]. Up to half of the stillbirths in low- and middle-income countries are carried to term (>36 weeks’ gestation) and weigh ≥ 2500g, suggesting that these stillbirths are potentially preventable [1]. 

While the epidemiology of stillbirths is not well-understood, the causes and risk factors for neonatal deaths are better known. Globally, the leading causes of neonatal mortality are preterm birth, infections and asphyxia [4]. In low- and middle-income countries, socio-demographic risk factors for neonatal deaths include maternal age, education, parity and access to antenatal and antepartum care [5-9]. 

Despite marked improvements in child survival, neonatal mortality constitutes 57% of the burden of child deaths in Bangladesh [10]. In Bangladesh, between 1990 and 2009, the under-5 mortality rate decreased from 148 to 52 per 1000 live births, and the infant (children < 12 months) mortality rate decreased from 102 to 41 per thousand [11]. However, this decrease in child mortality has not been accompanied by a comparable reduction in rates of neonatal (< 28 days) mortality, which was 30 per 1,000 live births in 2009 [11], a decline of 4% since 2000 [10], or of stillbirths, which declined by only 15% between 1975 and 2002 [12]. 

A recent study in a Dhaka slum identified birth asphyxia, sepsis and birth trauma as the main causes of neonatal mortality [13]. With 63% of the population living in rural areas, the majority of births in Bangladesh occur in rural areas where access to antenatal and obstetric care services is limited. Less than half (46%) of women living in rural Bangladesh report receiving antenatal care from medically trained personnel and only 18% report having a trained birth attendant (doctor, nurse or midwife) being present at delivery [14]. These factors likely contribute to the higher rates of stillbirths and neonatal deaths in Bangladesh. 

Socio-demographic and intrapartum risk factors for adverse pregnancy outcomes have been well-studied. Whether information readily available during mid-gestation can be used to identify high-risk pregnancies in low-income settings has, to our knowledge, not been considered. We used data collected in the context of a prospective cohort study evaluating the impact of an intervention aimed at improving maternal and child nutrition and infant development in rural Bangladesh to identify the maternal and antenatal factors associated with stillbirths and neonatal deaths.

## Materials and Methods

### Setting

Window of Opportunity is a prospective community-based nutrition and infant and young child feeding program being implemented by CARE in six countries, including Bangladesh, where it is known as *Akhoni Shomay*. *Akhoni Shomay* is being carried out in Karimganj, a rural sub-district of Kishoreganj, approximately 120 km north of Dhaka. Women living in a second sub-district, Katiadi, serve as controls for program evaluation purposes. This area is one of the poorest regions in the country.

### Recruitment

In the context of program evaluation activities, 2400 women who were residents of Karimganj or Katiadi were recruited into a prospective cohort study. Participants were recruited between January and October 2011. The timing of study recruitment was determined by financial and logistical considerations within the context of the community-based intervention it was designed to complement. Pregnant women were recruited in their 7^th^ month of gestation, with follow-up of their offspring scheduled to occur at 3, 9 and 16 months of age. Women who had resided in the study area for < 6 months at time of enrollment and/or having any chronic or congenital illness were excluded. As part of study follow-up, all child deaths are recorded and verbal autopsies are conducted. Stillbirths and neonatal deaths were identified at the time of the 3-month follow-up, and a verbal autopsy was conducted within 3-6 weeks of the death being identified. 

### Verbal autopsies

Bangladesh, like many other low-income countries does not have an established and reliable method of reporting deaths, especially child deaths. Knowing the leading causes of death is essential to informing public health policy so that the most appropriate and effective interventions can be implemented. Verbal autopsy has become an increasingly accepted method of assigning cause of death in these settings. As more efforts are being spent to ensure standardized administration of verbal autopsies, their reliability and validity is also improving. 

The International Standard Verbal Autopsy Questionnaire [15] was used to collect detailed information on clinical signs and symptoms during gestation, delivery and around the time of the child’s death. Information on care-seeking practices for the infant was also collected. The questionnaire includes closed and open-ended questions. Interviewers were trained in approaches to households reporting a child death. During the administration of the questionnaire, the mother/primary care-provider of the deceased child was also asked to narrate their experiences during the pregnancy, delivery, immediately after delivery, and the causes of illness that they believe led to the child’s death. Informed consent was obtained from the mother/care-provider of each child. An International Center for Diarrheal Disease Research, Bangladesh (icddr,b) clinician, experienced in verbal autopsy classification, assigned cause of death using International Classification of Diseases (ICD-10) [16]. A single cause was assigned to each stillbirth/neonatal death.

### Selection of cases and controls

Cases were defined as all stillbirths and all deaths that occurred within 28 days of live birth. Verbal autopsies were conducted between June 7, 2011 and March 16, 2012. Controls were a randomly selected sample from those children for whom the 3-month follow-up was completed. Four unmatched controls were selected for each case, as there is minimal marginal increase in statistical efficiency and precision beyond four controls per case [17,18]. 

### Variable derivation

Information on socio-demographic characteristics, including number of meals consumed per day in past 30 days, previous pregnancy outcomes, antenatal care and iron tablet/syrup consumption was abstracted from the questionnaire administered during 7^th^ month of gestation (at recruitment). History of bleeding and convulsions during pregnancy and delivery, place of delivery, type of birth and child’s gender was abstracted from the verbal autopsy questionnaire for cases and from the 3-month follow-up questionnaire for controls.

### Statistical analysis

Data were imported into Statistical Analysis Software (SAS), version 9.3 for analysis. Median (range) was calculated for continuous variable and frequencies and percentages were calculated for categorical variables. Socioeconomic status was assessed through an asset index score [19]. Information on household items (TV, telephone, furniture etc.), means of transportation (car, truck, boat etc.) and cattle was collected on the questionnaire administered at recruitment. Each asset was assigned a score equal to the inverse of the proportion of the study population that owned it. The asset index score was calculated as the sum of the scores for each individual asset owned by a study participant. 

Stillbirth and neonatal mortality rates were calculated. Associations between stillbirths and neonatal deaths (outcomes) and each of the variables of interest were assessed by way of odds ratios (OR). There was no evidence of collinearity among the predictor variables. A multivariable model for each of the outcomes was fitted using backward elimination. All maternal and antenatal variables considered to be associated with the outcomes at p < 0.2 were included. The p-value cut-off of 0.2 is considered adequate to prevent residual confounding in a multivariable model [20,21]. Statistical significance to remain in the final multivariable model was set at <0.05.

### Ethics

The parent prospective cohort study was approved by the Research Review Committee (RRC) and Ethical Review Committee (ERC) of International Center for Diarrheal Disease Research, Bangladesh (icddr,b). At the time of enrollment, written consent was taken from pregnant women. During this process, it was also mentioned that they would be followed-up when their offspring was 3, 9, and 16 months of age. They were also told that in the event of stillbirth or death of the child at any point during the study, a verbal autopsy would be conducted. 

At each follow-up, including verbal autopsy, verbal consent was obtained from the mother. This was documented by ticking a box in the questionnaire and showing it to her. Study participants were assured about the non-disclosure of information collected from them. They were also informed about the use of data for analysis and using the results of this study for policy planning regarding childhood malnutrition, as well as publication without disclosing the name or identity of their children. This consent procedure did not differ for participants < 18 years of age. RRC and ERC were satisfied with the voluntary participation, maintenance of the rights of the participants and confidential handling of personal information by the research personnel and approved this consent procedure. 

## Results

We recruited 2400 pregnant women, who reported 2414 deliveries, including 17 twin births. Two women were found to be not pregnant after recruitment and one woman had not delivered five months after recruitment. We successfully contacted 2317 women at the 3-month follow-up. Reasons for loss to follow-up included being unable to locate the mother (n = 62), refusal (n = 17) and maternal death (n = 1). These households were excluded from analysis.

At the 3-month follow-up, 112 stillbirths and neonatal deaths were recorded. Verbal autopsy questionnaires were completed for all 112 deaths. The median (range) interval between death and verbal autopsy was 116 (33 - 218) days. 

Of these deaths, 44 were stillbirths and 68 were neonatal deaths resulting in a stillbirth rate of 19 per 1,000 births and a neonatal mortality rate of 29 per 1,000 live births. Males comprised 60% of these deaths.

Of the stillbirths, 25 (57%) were classified as fresh. Of the neonatal deaths, 68% (n = 46) occurred during the first week of life. The leading cause of neonatal deaths was sepsis/pneumonia (48%), followed by birth asphyxia (35%) and preterm birth (19%). Causes of neonatal deaths are summarized in Table 1. 

**Table 1:** Specific causes of perinatal deaths in two sub-districts of rural Bangladesh, as assigned by verbal autopsy.

	**N (%)**
**Stillbirths**	44
Fresh	25 (57)
Macerated	19 (43)
**Neonatal deaths**	68
Birth asphyxia	24 (35)
Sepsis	19 (28)
Prematurity	13 (19)
Pneumonia	6 (9)
Other	6 (9)

Twelve twin pairs survived the neonatal period. For one set of twins, one of the twins was stillborn, while the other survived the neonatal period. For three sets, both twins died during the neonatal period. In a fifth set, one twin died during the neonatal period. 

Characteristics of stillbirths, neonatal deaths and their controls are summarized in Table 2. The proportion of mothers who received antenatal care was also similar across study groups. Compared to controls, mothers who experienced a stillbirth or neonatal death were less literate, were more likely to have a history of bleeding and eclampsia during pregnancy. Compared to controls, women who experienced a stillbirth or neonatal death were also more likely to have experienced prolonged labor and antepartum hemorrhage during delivery. The proportion of primaparous women was also similar across study groups. 

**Table 2:** Characteristics of stillbirths, neonatal deaths and survivors to age 3 months among pregnancies in Kishoreganj, Bangladesh.

	**Stillbirth (n = 44)**	**Neonatal death (n = 68)**	**Alive at 3 months (n = 448)**
**Socio-demographic characteristics of pregnant woman**			
Age in years			
15 - 24, %	50.0	45.6	51.3
25 - 34, %	45.4	50.0	43.3
> 35, %	4.6	4.4	5.4
Literacy			
Cannot read at all, %	47.7	45.6	33.0
Can read part of a sentence, %	18.2	7.4	18.5
Can read a complete sentence, %	34.1	47.1	48.4
<3 meals consumed per day (average over last 30 days) , %	4.5	20.6	11.6
Socioeconomic status (%)			
1^st^ quintile (lowest)	15.9	22.1	20.5
2^nd^ quintile	31.8	13.2	19.4
3^rd^ quintile	18.2	20.6	20.1
4^th^ quintile	20.5	19.1	20.1
5^th^ quintile (highest)	13.6	25.0	19.9
**Previous pregnancy outcomes**			
Primipara (%)	22.7	23.5	25.9
No prior adverse outcome (%)	47.7	41.2	52.2
Stillbirth or neonatal/infant death (%)	29.5	35.3	21.9
**Antenatal care**			
History of anemia (%)	20.5	14.7	-^a^
Received antenatal care (%)	52.3	42.6	43.1
Consumed iron tablets/syrup (%)	50.0	38.2	48.7
History of bleeding during pregnancy (%)	11.4	5.9	0.7
Eclampsia (%)	6.8	2.9	0.2
**Delivery care**			
Delivered at health facility (%)	36.4	13.2	16.5
Prolonged labor (%)	13.6	17.6	7.6
Emergency cesarean section (%)	15.9	2.9	8.3
Antepartum hemorrhage (%)	4.5	7.4	1.1
Convulsions during delivery (%)	6.8	1.5	1.3
**Fetus/Newborn**			
Child is male, %	61.4	58.8	49.5
Multiple births (%)	2.3	10.3	1.1

^a^ Follow-up questionnaire administered to survivors at 3-months of age did not ascertain this information

Table 3 summarizes the maternal and antenatal characteristics associated with stillbirths and neonatal deaths in our study population. Socioeconomic status was not associated with increased risk for either outcome. History of bleeding during pregnancy was strongly associated with increased risk of stillbirth (adjusted odds ratio 22.4 [95% confidence interval 2.5, 197.5]) was and with increased risk of neonatal death (adjusted odds ratio 19.6 [95% confidence interval 2.1, 178.8]), whereas consuming three or more meals a day was associated with better neonatal survival (adjusted odds ratio 0.4 [95% confidence interval 0.2, 0.8]).

**Table 3:** Socio-demographic and antenatal characteristics associated with stillbirths and neonatal deaths in rural Bangladesh – results of bivariable and multivariable regression analyses.

	**Stillbirth (n = 44)**				**Neonatal death (n = 68)**			
	**OR (95%CI)**	**P^a^**	**aOR (95%CI)**	**P^b^**	**OR (95%CI)**	**P^a^**	**aOR (95%CI)**	**P^b^**
**Socio-demographic characteristics of pregnant woman**								
Age	1.0 [1.0, 1.1]	0.47	-^c^		1.0 [1.0, 1.1]	0.06	-^d^	
Literacy		0.17		0.30		0.05	-^d^	
Cannot read at all	1.0 Reference				1.0 Reference			
Can read part of a sentence	0.9 [0.4, 2.3]		1.0 [0.4, 2.5]		0.3 [0.1, 0.9]			
Can read a complete sentence	0.5 [0.2, 1.1]		0.6 [0.3, 1.2]		0.7 [0.4, 1.2]			
≥3 vs. <3 meals consumed per day (average over last 30 days)	2.4 [0.5, 10.7]	0.25	-^c^		0.4 [0.2, 0.9]	0.03	0.4 [0.2, 0.8]	0.01
Socioeconomic status		0.31	-^c^			0.86	-^c^	
1^st^ quintile (lowest)	1.0 Reference				1.0 Reference			
2^nd^ quintile	2.5 [0.9, 6.8]				0.7 [0.3, 1.7]			
3^rd^ quintile	1.2 [0.4, 3.8]				0.9 [0.4, 2.1]			
4^th^ quintile	1.4 [0.5, 4.0]				0.9 [0.4, 2.1]			
5^th^ quintile (highest)	0.9 [0.3, 2.9]				1.2 [0.5, 2.5]			
**Previous pregnancy outcomes**		0.53	-^c^			0.12	-^d^	
Primiparous	0.9 [0.4, 2.1]				1.1 [0.5, 2.1]			
No prior adverse outcome	1.0 Reference				1.0 Reference			
Stillbirth or neonatal/infant death	1.5 [0.7, 3.3]				1.9 [1.0, 3.5]			
**Antenatal care**								
Received antenatal care (%)	1.4 [0.7, 2.8]	0.28	-^c^		0.9 [0.5, 1.6]	0.76	-^c^	
Consumed iron tablets/syrup (%)	1.0 [0.5, 2.0]	0.95	-^c^		0.6 [0.4, 1.1]	0.09	-^d^	
History of bleeding during pregnancy (%)	22.4 [2.5, 197.5]	<0.01	22.4 [2.5, 197.5]	<0.01	17.0 [1.9, 154.5]	<0.01	19.6 [2.1, 178.8]	<0.01
Eclampsia (%)	2.7 [0.4, 17.0]	0.28	-^c^		4.1 [0.8, 21.1]	0.10	-^d^	

OR, odds ratio; CI, confidence intervals; aOR, adjusted odds ratio

^a^ P-value resulting from bivariable logistic regression

^b^ P-value resulting from multivariable logistic regression

^c^ Not included in multivariable model as bivariable p-value > 0.2

^d^ Excluded from final multivariable model as p-value > 0.05

We also assessed the positive (PPV) and negative predictive values (NPV) of our multivariable models (see Table 4). For stillbirths, history of bleeding during pregnancy had a PPV and NPV of 83.3% and 81.8%, respectively. For neonatal deaths, history of bleeding during pregnancy and adequate maternal nutrition (≥3 meals/day) had a PPV and NPV of 80% and 81.6%, respectively. 

**Table 4:** Sensitivity, specificity, positive predictive value and negative predictive value of multivariable models identifying risk factors for stillbirth and neonatal death in rural Bangladesh.

	**Stillbirth^a^**				**Neonatal death^b^**			
**Variables**	**Sensitivity, %**	**Specificity, % **	**Positive predictive value, %**	**Negative predictive value, %**	**Sensitivity, % **	**Specificity, % **	**Positive predictive value, %**	**Negative predictive value, %**
History of bleeding during pregnancy only	11.4	99.4	83.3	81.8	-	-	-	-
<3 meals/day + NO bleeding during pregnancy	-	-	-	-	20.6	89.8	33.3	82.0
≥3 meals/day + NO bleeding during pregnancy	-	-	-	-	73.5	10.6	16.9	61.7
≥3 meals/day + history of bleeding during pregnancy	-	-	-	-	5.9	99.6	80.0	81.0

^a^ Stillbirth model only included history of bleeding during pregnancy

^b^ Neonatal death model included number of meals/day and history of bleeding during pregnancy

We also investigated whether delivery and antepartum characteristics were associated with neonatal death. Antepartum hemorrhage was most strongly associated with increased risk of neonatal death (adjusted odds ratio 11.8 [95% confidence interval 2.2, 62.9]), while cesarean birth was associated with improved newborn survival (adjusted odds ratio 0.2 [95% confidence interval 0.03, 0.9]).

## Discussion

Our study investigated the determinants of stillbirth and neonatal mortality in a community-based study in rural Bangladesh. Almost two-thirds (63%) of deaths in our population were fresh stillbirths or early neonatal deaths, indicating lack of access to quality obstetric care during labor and delivery. These deaths are potentially preventable if barriers to healthcare access for pregnant women are resolved. We also observed that antepartum hemorrhage was a significant risk factor for neonatal death, while consuming 3 or more meals per day during pregnancy was associated with improved neonatal survival in our population.

The main assigned causes of neonatal death in our population were birth asphyxia, sepsis and preterm birth. This order differs slightly from that reported by Lawn et al [4], who found preterm birth to be the leading cause of death among newborns, followed by infection and asphyxia. As mothers were recruited in their 7^th^ month of gestation, we know there were no very-early preterm births; but as follow-up was not conducted at time of birth, prevalence of later preterm birth cannot be determined. Therefore, preterm birth as cause of death may be an underestimate in our cohort. 

Working in Dhaka slums, Khatun et al [13] found birth asphyxia and sepsis to be the main causes of neonatal deaths in. With 35-42% of neonatal deaths attributed to it, birth asphyxia seems to be the leading cause of neonatal mortality in Bangladesh. Therefore, programs aimed at improving newborn survival in the country will need to include interventions that treat birth asphyxia.

Previous history of stillbirth is a well-established risk factor for recurrent stillbirth and other adverse outcomes in successive pregnancies [22,23]. We did not observe this relationship in our study population. It is very difficult, if not impossible to accurately determine the underlying causes of previous stillbirths and correct them, especially in a setting similar to ours. Therefore, interventions aimed at reducing stillbirth rates in Bangladesh and other low-income settings should be similar to those aimed at improving maternal and newborn survival in general. These should include early initiation of and more rigorous antenatal care and delivering at a health facility in the presence of a skilled birth attendant.

Approximately 60% of the stillbirths and neonatal deaths in our study were male. This is not unanticipated as it is well-established that boys are predisposed towards worse survival during the neonatal period compared to girls [24,25]. Other studies conducted in similar settings and looking at determinants of neonatal survival have observed the same [26-28]. 

In our population, a higher proportion of mothers of newborns who survived to 3-months of age were literate compared to those women whose off-spring were stillborn or died in the neonatal period. This is not surprising. The relationship between maternal literacy/education has been well-established in similar settings [29-32]. Therefore, educating mothers and primary care-givers of newborns will constitute an important avenue for interventions aimed at child survival. Such messages can be delivered through community health works and should include information on birth preparedness, including identification of a skilled delivery attendant, as well as a health facility and means of transport in case of obstetric complications.

Less than 20% of all participants included in our study delivered at a health facility, similar to that reported from other low-income settings [33]. A major cited reason for the high proportion of home deliveries in these settings is difficulty of access to healthcare facilities [34,35]. This was certainly true in our study setting. Kishoreganj is situated in the low-lying areas of Bangladesh and a significant portion of the land remains underwater for up to 8 months out of the year. Many households live on small “islands”, which are pockets of comparatively higher ground, manually reinforced with rocks, bamboo fences and sand-filled sacks to prevent erosion. Travelling a distance of only 10 km may take many hours as those wishing to leave the “islands” need to wait to for a passing boat to take them to an access road that connects to the major highway in Kishoregangj. The travel time by boat can be as long as 60 minutes, and travel on the road can add another 60 minutes. In the dry season, the distance travelled by boat has to be completed on foot, which takes even longer. This barrier to access may be a major reason for why more than 80% of the births in our study occurred at home. 

Being a twin is a risk factor for adverse pregnancy outcomes, especially in low- and middle income countries [36]. However, as there were only 17 pairs of twin births in the cohort (5 of which resulted in stillbirth or neonatal death) we could not assess the association of twinning with stillbirths or neonatal death for our population.

The higher proportion of cesarean births in children who were alive at three months of age (8% vs. 3% among those who died in the first 4 weeks of life) is indicative that there is some measure of medical oversight already in place that can identify high-risk pregnancies and convince the mother/family to deliver at a health facility. It is critical to remove the geographic barriers to access to care and ensure that health facilities in these areas are adequately equipped to deal with intrapartum complications that may arise during delivery.

About 18% of the stillbirths and neonatal deaths were due to preterm birth; a third of these may be prevented with the use of antenatal calcium supplementation to prevent pre-eclampsia and eclampsia [37]. Labor surveillance (including partograph) for the early detection of intra-partum complications can reduce early neonatal death by up to 40% [37]. Together, these two interventions might have prevented up to 22% of all stillbirths and neonatal deaths in our study.

Although there have been several studies assessing algorithms to predict risk of neonatal death based on presenting clinical signs [38,39], to our knowledge none have looked at the usefulness of maternal and antenatal characteristics in predicting risk of stillbirth and/or neonatal death. Our models, which include information readily available during mid-pregnancy, do well in accurately identifying pregnancies at higher risk of adverse outcomes. Community health workers in low-income settings, for example, can easily identify women who have had bleeding during gestation and counsel them to seek antenatal care and skilled care during delivery. This avenue may constitute an important addition to the public health arsenal improving newborn survival. 

The median interval between a death and verbal autopsy in our setting was 17 weeks. This allowed for some period of mourning to have occurred, but is a source of potential recall bias in our study. For controls, data on delivery variables was obtained during the 3-month follow-up questionnaire. Given that cases in our study were newborns that died, maternal/primary care-giver recall may be differential between the cases and controls. Second, as mentioned before, one icddr,b clinician assigned cause of death after reviewing verbal autopsies. As no other independent assessment was done, validity of these assignments cannot be determined. Furthermore, only one cause was assigned to each death. Therefore, role of underlying causes, such as undernutrition was not determined.

In conclusion, the majority of stillbirths and neonatal deaths in our setting were potentially preventable with access to quality obstetric care during labor and delivery. Furthermore, maternal and antenatal characteristics can be used to identify newborns at higher risk of death. Programs designed to improve newborn survival in Bangladesh and similar settings should include a mechanism which identifies high risk pregnancies and births well before the expectant mother goes into labor. These programs should also include interventions aimed at removing barriers to healthcare access for pregnant women and their newborns.
